# Month of Birth and the Incidence of Multiple Sclerosis in Southern Iran

**Published:** 2014-03

**Authors:** Alireza Nikseresht, Maryam Sharifian, Alireza Hamidian Jahromi

**Affiliations:** 1 Department of Neurology, Motahari Clinic, Shiraz University of Medical Sciences, Shiraz, Iran;; 2 Student Research Committee, Shiraz University of Medical Sciences, Shiraz, Iran;; 3 Department of Surgery, Louisiana State University Health Sciences Center, Shreveport, LA, USA

## Dear Editor,

There are some previous studies that have proposed that seasons of birth are a potential risk factor for the development of multiple sclerosis (MS) later in life. Recent studies on the seasonal pattern of MS patients have shown a potential spring peak and an autumn nadir.^1^ One possible explanation is the decreased exposure to sunlight in winter, which leads to low vitamin D levels during pregnancy.^2^

In contrast, two recent studies that were performed in non-European populations by Givon et al.^1^ and Fragoso et al.^3^ did not find any significant correlation between the month of birth (MOB) and the risk of MS involvement later in life.

In this study, we examined the relationship between the MOB and season of birth (SOB) and the risk of MS later in life in a southern Iranian population. A total of 1558 patients (1020 women and 538 men, aged between 15 and 65 years) from southern Iran with a definite MS diagnosis according to McDonald’s criteria were evaluated for inclusion in a retrospective case-control study. The medical records of these patients were obtained from our Outpatient Clinic in Shiraz, Fars. Two thousand one hundred individuals (1600 women and 500 men) were randomly selected from the normal population of Fars Province (visitors to the Cardiology, Urology, and Surgery Wards in Nemazee Hospital and Faghihi Hospital, Shiraz, Fars) and were matched with a case group by age and sex. Control group individuals with any history of autoimmune diseases or a history of MS in themselves or their families were excluded from the control group. 

The study was approved by the institutional Review Board and the Ethics Committee of Shiraz University of Medical Sciences. Descriptive statistics and Chi-squared test by SPSS software (version 17) were used for statistical analysis. A P<0.05 was considered statistically significant. 

Our results showed that most women were born in March [98 (9.6%) in the cases and 70 (4.3%) in the controls] and April [134 (13.1%) in the cases and 85 (5.8%) in the controls].

 In the males, the rate of birth among the patients with MS was significantly higher than that in the controls in March [30 (5.6%) in the patients and 10 (1.2%) in the controls] and April [126 (23.7) in the patients and 75 (8.8%) in the controls] (P<0.05). Moreover, it seems that the rate of birth was significantly lower in the case group in August [18 (3.4%) in the patients and 70 (8.1%) in the controls] and December [20 (3.7%) in the patients and 75 (8.7%) in the controls] (P<0.05). 

Considering all the patients, the rate of birth among patients with MS was significantly higher than that in the controls in March [128 (8.2%) in the patients and 80 (3.2%) in the controls], April [260 (16%) in the patients and 160 (6.4%) in the controls], and October [144 (9.2%) in the patients and 105 (4.2%) in the controls] (P<0.05). 

No significant difference was detected between the cases and controls regarding the time of birth in the different seasons of the year.

Complex disorders such as MS have no single cause but result from a combination of genetic and environmental factors and their interactions. Several studies have investigated the effect of the MOB in MS patients with MS. Some studies have demonstrated that there is no relation between the MOB and the risk of MS.^3^^,^^4^ However, Dobson et al.^5^ in their meta-analysis demonstrated a significant excess of MS risk in those born in April.

Some studies have indicated that while the MOB effect is more prominent in high-risk areas for MS, especially in areas with low sunlight exposure, this effect seems to be negligible or non-existent in areas with high sunlight exposure. This may provide a good explanation for the discordant results of the studies on the possible association between the MOB and MS in different parts of world with various levels of sunlight exposure.^6^ Moreover, the theory of sun exposure and the possible protective role for vitamin D concentrations during pregnancy or early life of the newborn may further explain these findings.^4^

Our study is one of the first studies to assess the association between the MOB and MS incidence in an Iranian MS population (southern Iran), and the results are similar to those reported by many previous studies. 
